# The Royal Naval Medical Service

**Published:** 1918-09-07

**Authors:** 


### THE ROYAL NAVAL MEDICAL SERVICE.

THOUGH infinitely smaller than the Royal Army Medical Corps, the Senior Service has claimed several hundred men from the ranks of civilian practice, to augment the regular officers on the larger ships, and to take sole medical charge on the numerous smaller craft that are now being launched in increasing numbers.

The system of entry is a good one. Nearly all officers who have joined during the last two years have already served for six months or more as surgeon probationers, and as such have already had a taste of what naval life under active service conditions means. The Navy, as befits its ancient traditions, is naturally far more punctilious in matters of etiquette and precedence than our new and democratic Army has any need to be, and it is well that the embryo surgeon should learn something of naval routine in the comparatively easy-going wardrooms of destroyers and mine-sweepers than be left to take his chance amid the rigid decorum of a flagship or cruiser.

On entry the surgeon is first posted to one of the three permanent naval hospitals, Haslar, Chatham, or Devonport, where he learns to recognise the innumerable "chits," certificates, requisitions, surveys, and returns, in the filling up of which so much of his time in the future will be spent. The life here is pleasant, every opportunity is afforded for recreation and social intercourse, and for a time, at all events, the stern business of war can be almost forgotten. But all too soon, alas! this pleasant interlude is ended. An intimation is received that he must proceed to —— where he will join H.M.S. ——, and for two years, at least, the pleasant paths of the "shore billet" will be forsaken for the grey waters of the North Atlantic or the blue waves of the Mediterranean. At the expiration of this period he can apply to be appointed to a shore hospital for a twelvemonth, an excellent provision which prevents a man becoming too stale and behindhand with his work.

Of the life afloat there is little to be said. The duties of ships' surgeons are not onerous, for the seaman is a healthy animal, and with rare exceptions epidemic disease is unknown. Accidents are from time to time inevitable, but any injury or disease that is likely to last more than a few days is at once sent to the depôt ship or nearest shore hospital for treatment. It is a curious and interesting fact that the only times when the surgeon has his hands full are after the ship's company has been ashore on leave. In stations where there is no shore leave for months on end the health of the crews remains excellent and the ship's surgeon has a quiet time.

In action, of course, it is a different story, and the Honours lists have shown how finely surgeons, especially on the smaller craft, have carried out their duties, under conditions of appalling difficulty. From aid posts at all quarters of the ship, wounded are brought four and five decks down to a small dressing station and operating theatre where, protected by thick armour-plating, the surgeons and their assistants perform such operations as are possible and make the wounded as comfortable as circumstances permit. The surgeon sees nothing of the action; indeed, his may be said to be a position of some peril, for in the event of the ship foundering, escape would be well-nigh impossible.

The life of the naval surgeon is, like many another, a time of unavoidable tedium, varied by moments of acute excitement. But the hours do not hang too heavily, for many duties are trusted to the "Doc," the keeping of mess accounts, wine and tobacco lists, censoring letters, all of which make him feel as though his existence on board was justified. The "N.O." (naval officer) is a philosophic fellow, and whilst waiting for the supreme moment when he shall at last come to grips with the wily Hun, he contrives to make his mess as cheery a place as food rations and active service will allow.

